# Microfilaria concomitant with metastatic deposits of adenocarcinoma in lymph node fine needle aspiration cytology: A chance finding

**DOI:** 10.4103/0970-9371.70759

**Published:** 2010-04

**Authors:** Sachin S Kolte, Rahul N Satarkar, Pratibha M Mane

**Affiliations:** Department of Pathology, Mamata Medical College, Khammam, Andhra Pradesh, India; 1 Department of Microbiology, Mamata Medical College, Khammam, Andhra Pradesh, India

Sir,

Filariasis is a major health problem of the tropics and subtropics, commonly seen in countries like India, China, Indonesia, Africa, and the Far East. The disease is endemic all over India.[1] In India, *Wuchereria bancrofti* accounts for 90% of the total filarial infections.[2] Despite its high incidence, it is infrequent to find microfilariae in fine needle aspiration cytology (FNAC) smears and body fluids. Microfilariae have been observed as coincidental findings with other inflammatory conditions and neoplastic lesions.[3–6] Microfilariae at the site of the primary malignant tumor have been reported, but their coexistence with metastatic deposits have not been reported so far. We present here a rare case in which microfilariae were encountered in the fine needle aspiration of the supraclavicular lymph node along with secondary deposits from mucinous papillary adenocarcinoma.

A 45-year-old male presented in the outpatient department with supraclavicular and cervical lymphadenopathy since 3 months. The left supraclavicular lymph node was of size 3cm×3cm and the left cervical lymph node was of size 2cm×2cm. The patient was referred to the pathology department for FNAC of the left supraclavicular lymph node. Microscopic examination showed highly cellular smears. Epithelial cells were seen arranged in groups, acinar pattern, and scattered singly. Cells were fairly uniform in size with round, hyperchromatic nuclei [Figure 1 lower inset]. Mucoid material was seen in the background. Along with these tumor cells, were noted sheathed microfilariae of *W. bancrofti* having multiple, coarse, discrete nuclei extending from the head to the tail, except in the small terminal portion of the caudal end [Figure 1 upper inset]. Based on the above findings, a diagnosis of metastatic deposits from adenocarcinoma with microfilariae of *W. bancrofti* was offered. The peripheral blood smear did not reveal any microfilariae. The patient was advised chest radiographs, which revealed a mass lesion in the upper lobe of the left lung.

**Figure 1:**
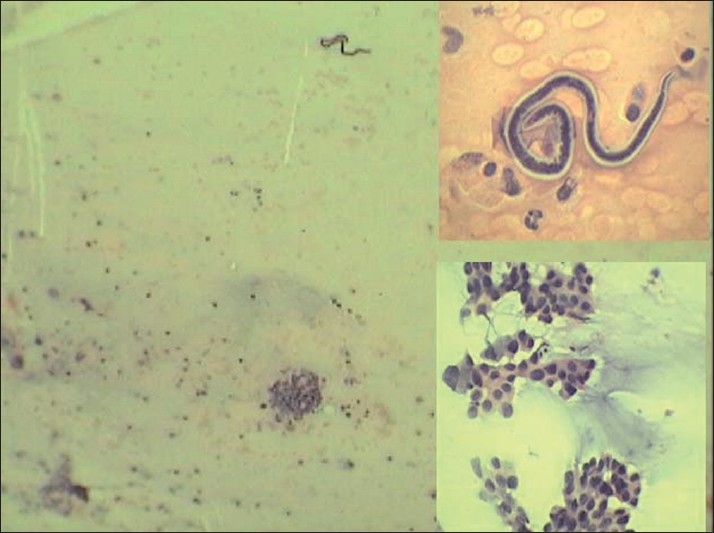
Fine-needle aspiration cytology of the lymph node showing tumor cells along with microfilaria (PAP ×40). Inset (upper) showing sheathed microfilaria with inflammatory cells. Inset (lower) showing tumor cells over a mucinous background (PAP, ×400)

It is estimated that about 600 million people are living in areas endemic for lymphatic filariasis in the Southeast Asian region. There are about 60 million people infected in the region and about 31 million people have clinical manifestation of the disease.[1]

Filariasis is a major public health problem in India. Heavily infected areas are found in Uttar Pradesh, Bihar, Jharkhand, Andhra Pradesh, Orissa, Tamilnadu, Kerala, and Gujarat. The disease manifestation ranges from none to both acute and chronic manifestations.[1]

It is transmitted by the Culex mosquito and is caused by two closely related nematodes, *W. bancrofti* and *Brugia malayi*, which are responsible for 90% and 10% of the cases, respectively.[2] Infective larvae penetrate the feeding wound in the skin, enter the lymphatics and travel to the lymph nodes. After maturation in a few months, they develop into white, thread-like adult worms and survive for several (10–18) years in the lymph nodes. Once fertilized, the female discharges several thousand microfilariae (150–300-*μ* long), which dwell in the peripheral blood for 5–10 years.[2]

Despite the high incidence of filariasis, microfilaria in FNAC is not a very common finding. There have been reports of single or small number of cases of microfilaremia at various sites such as lymph node, breast lump, bone marrow, bronchial aspirate, nipple secretions, pleural and pericardial fluid, ovarian cyst fluid, and cervicovaginal smears.[2]

Microfilariae and adult filarial worms have occasionally been detected in association with neoplastic lesions in cytological smears. On searching the literature for microfilaria with malignant neoplasm, we have found some case reports describing coexistence of microfilaria with primary malignant tumor.[3–5]

We have not found any case of microfilaria with secondary deposits. The presence of microfilariae along with neoplasms is generally regarded as a chance association.[5]

In the present case, we feel that the presence of microfilaria in association with the metastatic deposits is an incidental finding and that the patient was harboring subclinical filariasis when the tumor metastasized. No microfilariae were detected in the peripheral smears. Our case illustrates the coexistence of microfilariae with secondary deposits of adenocarcinoma, which is a rare finding. This also highlights the importance of screening smears for parasites even in the absence of clinical symptoms, particularly in highly endemic areas.

## References

[1] Park K (2005). Park’s Textbook of Preventive and Social Medicine.

[2] Chowdhary M, Langer S, Aggarwal M, Agarwal C (2008). Microfilaria in thyroid gland nodule. Indian J Pathol Microbiol.

[3] Varghese R, Raghuveer CV, Pai MR, Bansal R (1996). Microfilariae in cytologic smears: A report of six cases. Acta Cytol.

[4] Sinha BK, Prabhakar PC, Kumar A, Salhotra M (2008). Microfilaria in a fine needle aspirate of breast carcinoma: An unusual presentation. J Cytol.

[5] Gupta S, Sodhani P, Jain S, Kumar N (2001). Microfilariae in association with neoplastic lesions: Report of five cases. Cytopathology.

[6] Gupta K, Sehgal A, Puri MM, Sidhwa HK (2002). Microfilariae in association with other diseases. A report of six cases. Acta Cytol.

